# Microbial Dynamics in a Musalais Wine Fermentation: A Metagenomic Study

**DOI:** 10.3390/foods14152570

**Published:** 2025-07-22

**Authors:** Yongzeng Pei, Mengrong Chen, Qiling Chen

**Affiliations:** College of Food Science and Pharmacy, Xinjiang Agricultural University, Nongda East Road 311, Urumqi 830052, China; puwua12138@163.com (Y.P.); acc1408726@163.com (M.C.)

**Keywords:** spontaneous/wild fermentation, Musalais wine, microbial diversity, metagenomics, volatile compounds

## Abstract

This study provides a comprehensive analysis of the microbial dynamics involved in the fermentation process of traditional Musalais wine, an intangible cultural heritage of Xinjiang. Utilizing metagenomic sequencing, we identified 2894 microbial species, of which 494 persisted throughout the fermentation process. *Saccharomyces cerevisiae* was the dominant species, with its prevalence increasing from 97.35% in the early phase to 99.38% in the mid phase, before slightly decreasing to 98.79% in the late phase. Additionally, 24 non-*Saccharomyces* yeast species, including *Hanseniaspora uvarum*, *Lachancea thermotolerans*, and *Torulaspora delbrueckii,* were detected. Common species associated with other fermented foods, including *Wickerhamomyces anomalus*, *Kluyveromyces marxianus*, *Saccharomyces eubayanus*, and *Zygosaccharomyces parabailii*, were also identified. Notably, species not previously used in food fermentation, such as *Saccharomyces jurei*, *Sodiomyces alkalinus*, *Vanrija pseudolonga*, and *Moesziomyces antarcticus*, were also identified in this study. Furthermore, the Kyoto Encyclopedia of Genes and Genomes (KO) and Gene Ontology (GO) revealed notable variations in metabolic pathways and enriched functional genes. In addition, a total of 82 volatile compounds were detected in the final product, with higher alcohols (60.12%), esters (37.80%), and organic acids (1.80%) being the most prevalent. These results offer important insights into microbial interactions and their influence on Musalais wine quality, laying the groundwork for optimizing the fermentation process.

## 1. Introduction

Musalais wine, primarily crafted from Hetianhong grapes (*Vitis vinifera* L.), undergoes a meticulous process involving pressing, boiling, and filtering to produce the fermentation broth. This broth is then cooled and allowed to ferment naturally over a period of approximately 45 days. Recognized as the only traditional wine in China, Musalais wine represents an intangible cultural heritage originating from the Xinjiang region, with a historical lineage that spans over two millennia and has been preserved to the present day [1]. Presently, the main production area is Awati County, which is home to over 200 traditional workshops. Unlike conventional wine production, Musalais wine involves the extended boiling of grape juice before fermentation, imparting a distinctive caramel aroma and a pronounced alcoholic fragrance. Despite advancements in modern production techniques, some traditional workshops continue to use clay jar fermentation methods, while large-scale modern enterprises, such as Miandu Company, typically employ stainless steel tanks for mass production.

During spontaneous wine fermentation, microorganisms like *Saccharomyces*, *Tatumella, Hanseniaspora, Lactobacillus*, and *Lachancea* play crucial roles in shaping the wine’s flavor by producing specific volatile compounds. *Saccharomyces* is involved in glycolysis and pyruvate metabolism, generating ethanol and other metabolic precursors. *Lactobacillus* and *Lachancea* contribute to lactic acid metabolism, while *Tatumella* aids in amino acid, fatty acid, and acetic acid metabolism, forming esters [2]. Studies on Marselan and Merlot wines have revealed that these microorganisms significantly affect the wines’ physicochemical properties and enhance fruity, floral, and typical aromas through increased diversity and levels of aromatic compounds [3,4]. Microbial diversity is key to wine’s aroma, flavor, and quality, and understanding this can enhance fermentation practices for better wine.

To date, microbial diversity in Musalais wine has been explored through culture-dependent isolation coupled with phenotypic identification [5]. However, the inability to profile unculturable taxa and the labor-intensive processes of these traditional methods present significant limitations, restricting the comprehensive understanding of the microbial consortia’s architecture and the dynamic changes in their abundances during fermentation.

Notably, the incorporation of metagenomic sequencing into traditional food fermentation research has introduced a significant paradigm shift [6,7], thereby transforming the analysis landscape for microbial communities [8,9]. Studies have shown that this technological integration has facilitated high-resolution decoding of collective microbial genomes, allowing for the concurrent characterization of community architecture, taxonomic stratification, and functional gene networks via next-generation sequencing platforms [10,11].

This study aimed to investigate the dynamic changes in the microbial community during the multistage natural fermentation process of Musalais wine, addressing the limitations inherent in previous culture-dependent methodologies by employing metagenomic sequencing. The findings of this study may offer essential insights into the role of microorganisms in the flavor development of Musalais wine, thereby providing a research basis for developing specialized industrial strains, flavor regulation, standardized production processes, and technical criteria for Musalais wine products.

## 2. Materials and Methods

### 2.1. Experimental Materials

The experimental Musalais wine samples were collected from the Miandu Musalais Limited Liability Company, located in Awati County, Aksu Prefecture, within the Xinjiang Uygur Autonomous Region of China. The fermentation process was carried out in stainless steel tanks, with the temperature meticulously maintained at 20 °C (68 °F) through a temperature control system. Each tank had a capacity of 30 tons.

#### 2.1.1. Detection Methods for Fundamental Physical and Chemical Parameters

The quantification of soluble solids in Musalais wines was conducted using a handheld refractometer (Shanghai Jiahang Instrument Co., Ltd., Shanghai, China). In contrast, the fundamental compositional parameters of Musalais wines, including alcohol content and reducing sugars, were assessed using a WineScan (FT 120, FOSS Analytical, Hillerød, Denmark) rapid-scanning infrared Fourier-transform spectrometer, integrated with FOSS WineScan software version 2.2.1 (Foss Electric, Hillerød, Denmark).

#### 2.1.2. Wine Sample Collection and Processing

Musalais wine samples were collected from three distinct fermentation stages—early, middle, and late—with each stage represented by three replicates, culminating in a total of nine samples. Following collection, the wine samples were subjected to centrifugation at 8000 rpm to separate the microbial pellets. DNA was subsequently extracted from these microbial pellets for metagenomic sequencing.

### 2.2. DNA Extraction and Illumina-Based Sequencing

In accordance with the manufacturer’s guidelines, genomic DNA was extracted utilizing the HiPure Bacterial DNA Kits (Magen, Guangzhou, China). The quality of the extracted DNA was subsequently evaluated using both the Qubit and Nanodrop instruments (Thermo Fisher Scientific, Waltham, MA, USA). The genomic DNA that met quality standards was then fragmented to a size of 350 base pairs, followed by end-repair, A-tailing, and adaptor ligation, employing the NEBNext^®^ Ultra™ DNA Library Prep Kit for Illumina (New England Biolabs, Ipswich, MA, USA) as per the specified protocol. The resulting libraries were purified using the AMPure XP system (Beckman Coulter, Brea, CA, USA), and their size distribution was assessed using the 2100 Bioanalyzer (Agilent Technologies, Inc., Santa Clara, CA, USA). Quantification was conducted via real-time PCR. Finally, the samples were sequenced using paired-end 150 bp reads on the NovaSeq X Plus platform (Illumina, San Diego, CA, USA).

### 2.3. Bioinformatic Analysis

#### 2.3.1. Assembly, Gene Prediction, and Gene Catalog

The raw Illumina data were filtered with FASTP (v0.18.0), and the following reads were removed: reads with ≥10% unidentified nucleotides, ≥50% low-quality bases, and those aligned to barcode adapters. The clean reads were assembled using MEGAHIT (v1.2.9) (k-mer range: 21–141) [12]. Next, contigs of >500 bp were analyzed to predict genes using MetaGeneMark (v3.38) [13]. Predicted genes with ≥300 bp were pooled and deduplicated with CD-HIT (v4.6) [14] at ≥95% identity and 90% coverage. Finally, reads were aligned to the selected genes through Bowtie (v2.3.5.1) [15] to count reads to obtain the final gene catalog, which included non-redundant genes with >2 reads.

To quantify the abundance of genes, alignment, reassignment, and calculation were performed. Initially, reads were aligned to unigenes through Bowtie (v2.2.5) and then reassigned via PathoScope (v2.0.7) [16], which is known for generating highly sensitive profiles under typical metagenomic sequencing conditions. Finally, the relative abundance of each gene was calculated using the formula Gi = (Ri/Li)/$\sum_{j=1}^{n}$ (Rj/Lj), where G_i_ is the relative abundance of gene i in the sample S; L_i_ is the length of gene i; and R_i_ denotes the number of times gene i can be detected in the sample S (the number of mapped reads).

#### 2.3.2. Taxonomic Annotation

Herein, high-quality sequence reads were utilized to construct a taxonomic profile employing Kaiju (v1.10.1) [17]. The predicted genes were subsequently aligned to the sequences extracted from the National Center for Biotechnology Information non-redundant genome databases using Diamond (v0.9.25) [18], following analysis with MEGAN (v6.25.10) [19] to estimate the taxonomic compositions. For this analysis, the weighted Lowest Common Ancestor algorithm was applied.

#### 2.3.3. Analysis of Differences in Species or Function

Indicator species analysis is generally used to identify differential markers between different groups. First, the R labdsv package (v2.1-0) (https://cran.r-project.org/web/packages/labdsv/index.html accessed on 19 June 2024) was used to calculate the indicator value of each species in each group. Then, a 10 × 10 cross-validation was used to assess differential species by default. Welch’s *t*-test and Wilcoxon rank-sum test were used for non-parametric testing of two independent samples. ANOVA was used for differential testing of three or more independent samples. Box plots were used to display differential species, genes, and KOs among comparison groups by R stats package. A Venn analysis was performed using the VennDiagram package in the R programming environment [20] (https://cran.r-project.org/web/packages/venn/index.html accessed on 19 June 2024), and an upset plot was generated using the UpSetR package (v1.4.0) [21] to identify unique and shared species or functions. The intergroup comparisons of species/functions were performed using Welch’s *t*-test and the Wilcoxon rank-sum test, employing the Vegan package in R. For multigroup comparisons of species/functions, analysis of variance was employed, utilizing the Vegan package in R (v3.6.2).

#### 2.3.4. Alpha Diversity Analysis

The Chao1 and Shannon indices were computed using the R programming language (https://www.r-project.org/). The Chao1 indices specifically assess species richness, where higher values indicate greater diversity. In contrast, the Shannon indices provide a comprehensive evaluation of both species’ richness and evenness, with higher values signifying increased evenness. To compare the Chao1 and Shannon indices among the three fermentation stages, the Kruskal–Wallis H test and Tukey’s HSD test were utilized, also via the R stats package (v1.12).

#### 2.3.5. LEfSe Analysis

To identify differentially abundant microbial taxa and functions across fermentation stages, we used LEfSe (v1.0), a statistical tool for detecting significant differences among groups. The analysis involves three steps: using the Kruskal–Wallis test (*p* < 0.05) to find features with significant abundance differences, applying the Wilcoxon test for subgroup consistency, and calculating the effect size (LDA Score) to quantify feature contributions to group differences (significant if |LDA| > 2).

#### 2.3.6. Annotation Steps for KEGG (KO) and GO

After quality control and host sequence removal, we aligned the sequences against the UniProt protein database utilizing DIAMOND in conjunction with the HUMAnN3 software [22]. We excluded any reads that did not meet the alignment criteria, adhering to the default HUMAnN3 alignment parameters: translated_query_coverage_threshold set at 90.0, prescreen_threshold at 0.01, evalue_threshold at 1.0, and translated_subject_coverage_threshold at 50.0. Subsequently, we computed the relative abundance of each UniRef90 protein. Additionally, we employed the HUMAnN script, humann_renorm_table, to determine relative abundance based on the initial abundance data. By leveraging the correspondence between UniRef90 identifiers and those of various functional databases, such as KEGG and GO, we aggregated the abundance of genes (UniRef90) associated with the same function to derive the relative abundance of functions within the respective functional databases.

#### 2.3.7. Redundancy Analysis (RDA)

Redundancy analysis (RDA) was performed to assess the relationship between microbial community features and environmental factors using Wekemo Bioincloud [23]. Permutation tests (*n* = 999) confirmed model significance.

#### 2.3.8. Microbial Co-Occurrence Network

The co-occurrence patterns of microbial communities in Musalais wine across three fermentation stages were analyzed using Spearman’s rank correlation coefficients. Statistically significant co-occurrence events were identified based on robust correlations (|R| > 0.6, *p* < 0.05). The resulting co-occurrence network was subsequently visualized utilizing Gephi (v0.9.6).

### 2.4. Analysis of Volatile Compounds in Musalais Wine

The analysis for volatile compounds of Musalais wine was conducted through gas chromatography (GC)–mass spectrometry (MS) using an SH-Rxi-5Sil MS capillary column with detector and injector temperatures set at 230 °C and 250 °C. The oven temperature was initially set at 40 °C for 3 min and increased to 140 °C at a rate of 2 °C/min, followed by an increase to 270 °C at a rate of 7.5 °C/min, where it was held for 6 min. Helium (99.999% purity) was used as the carrier gas and set at a flow rate of 1.78 mL/min in the splitless mode. Electron ionization was performed at 70 eV, with ion source and transfer line temperatures set at 230 °C and 270 °C. MS readings were recorded in the full scan mode (*m*/*z*: 30–450). Each wine sample (3 mL) was placed in a 15 mL vial with a stir bar and mixed with 0.6 g of NaCl and 5 μL of 4-octanol. Thereafter, the tube was sealed, and the mixture was equilibrated at 45 °C for 15 min. A 65 μm PDMS/DVB fiber was exposed to the headspace for 45 min at 45°C, 5 mm above the liquid. Thermal desorption occurred in the GC injector for 5 min with data collection [24].

## 3. Results

### 3.1. The Basic Physical and Chemical Indicators of the Musalais Wine

**Figure 1:**
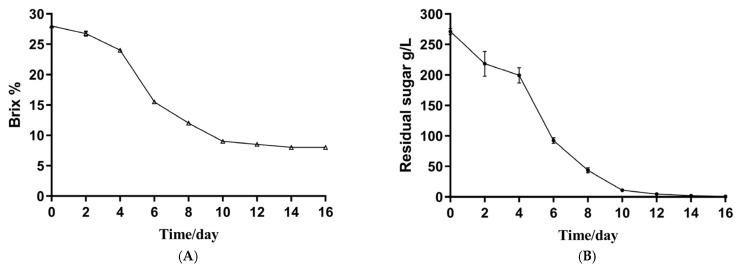
Investigation of physicochemical parameters of Musalais wine across various fermentation stages. (**A**) Measurement of Brix value; (**B**) assessment of residual sugar. Each sample has three parallel treatments.

The fermentation profile of Musalais wine is depicted in Figure 1. Specifically, Figure 1A illustrates the Brix sugar level data. In the production process, the termination of fermentation is frequently determined by assessing the Brix sugar level, with fermentation being deemed complete when this level stabilizes at 8. Figure 1B presents the residual sugar content in Musalais wine. The alcoholic fermentation of Musalais wine typically concludes around the 15th day of the fermentation process. For metagenomic sequencing, fermented wine samples were selected from days 1, 5, and 10 of the fermentation period.

### 3.2. Analysis of Microbial Community Structure at Different Fermentation Stages of Musalais Wine

#### 3.2.1. Alpha Diversity Analysis of Microorganisms in Musalais Wine Fermentation

**Figure 2:**
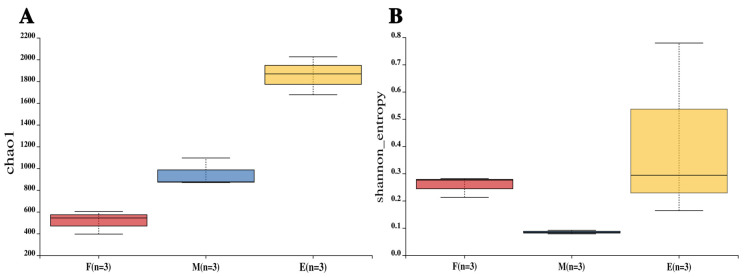
Alpha diversity indices of Musalais wine samples at different fermentation stages. (**A**) Chao1 index; (**B**) Shannon index. Note: F: early-fermentation period, M: middle fermentation period, E: late-fermentation period.

Herein, the Chao1 index (Figure 2A) and Shannon index (Figure 2B) were the key alpha-diversity metrics in the analysis and effectively characterized the richness and diversity of microbial communities. Notably, significant differences were found in both composition and diversity of microbial communities in Musalais wine samples during various fermentation stages. Furthermore, prolonged fermentation duration correlated with a progressive increase in microbial abundance, and microbial diversity exhibited an initial decrease followed by a subsequent elevation.

#### 3.2.2. Species Diversity Analysis of Microorganisms at Different Fermentation Stages of Musalais Wine

**Figure 3:**
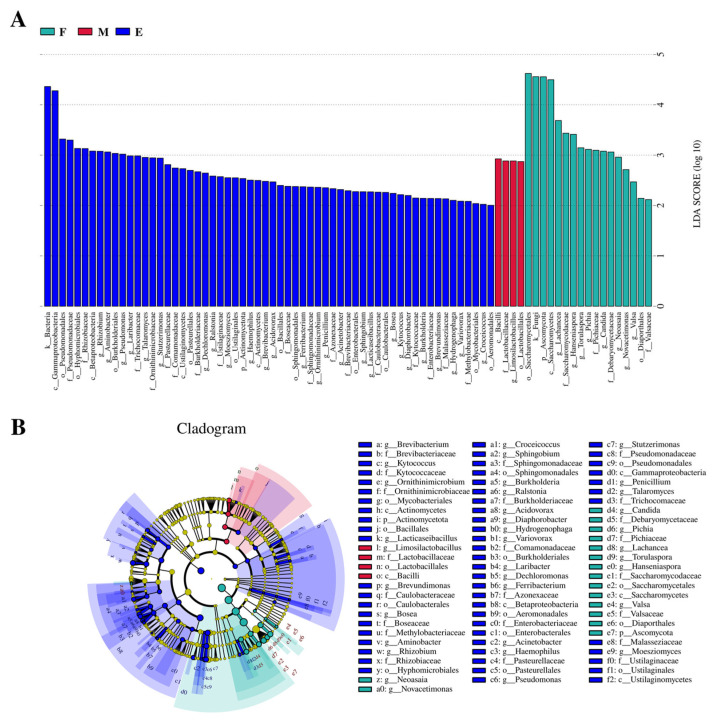
Linear discriminant analysis effect size (LEfSe) analysis delineating phase-specific microbial biomarkers in Musalais wine fermentation. (**A**) LEfSe histogram. (**B**) LEfSe analysis of the phylogenetics. Note: F: early-fermentation period, M: middle fermentation period, E: late-fermentation period.

In total, 79 microbial taxa at different taxonomic levels, with distinct microbial communities, were found to exhibit significant variations across different stages of Musalais wine fermentation (Figure 3). In the early-fermentation stage, genera, such as *Valsa*, *Hanseniaspora*, *Pichia*, *Candida*, *Torulaspora*, and *Neoasaia*, were identified as the differential genera. In contrast, during the mid-fermentation phase, the genus *Limosilactobacillus* was the predominant differential genus. In the late-fermentation stage, various differential genera were identified, including *Brevibacterium*, *Kytococcus*, *Ornithinimicrobium*, *Lacticaseibacillus*, *Brevundimonas*, *Bosea*, *Aminobacter*, *Rhizobium*, *Sphingobium*, *Burkholderia*, *Ralstonia*, *Acidovorax*, *Diaphorobacter*, *Hydrogenophaga*, *Variovorax*, *Laribacter*, *Dechloromonas*, *Ferribacterium*, *Acinetobacter*, *Haemophilus*, *Pseudomonas*, *Penicillium*, *Talaromyces*, and *Moesziomyces*.

At the phylum level, the differential microorganisms identified during the early stages of fermentation were primarily associated with Ascomycota. In contrast, during the mid-fermentation phase, Firmicutes was the most predominant phylum. In the late-fermentation stage, the differential microorganisms were mainly distributed within the phyla Actinomycetota and Proteobacteria. Overall, these findings reveal notable dynamic alterations in the microbial community structure throughout Musalais wine fermentation, with distinct compositional shifts corresponding to each fermentation stage.

#### 3.2.3. Microbial Community Structure Analysis Based on Taxonomic Levels

**Figure 4:**
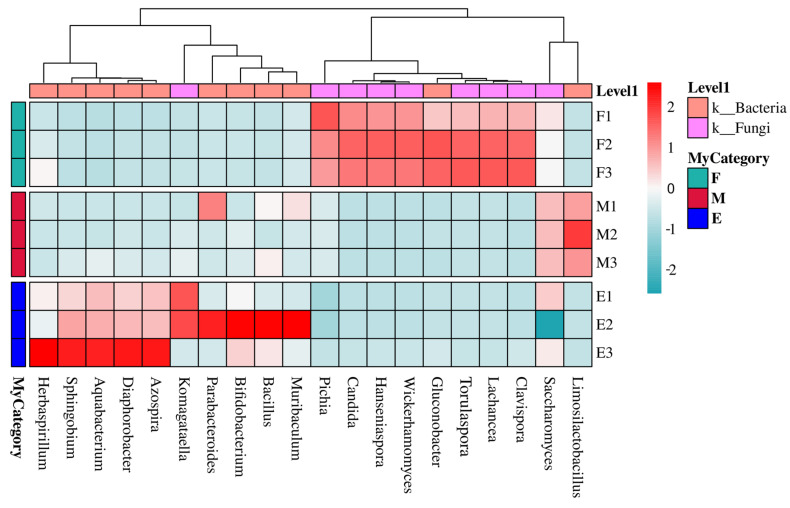
Taxonomic profiling of microbial communities at the genus level and temporal variations in relative abundance across Musalais wine fermentation stages. Note: F: early-fermentation period, M: middle fermentation period, E: late-fermentation period.

The top 20 enriched microbial genera during Musalais wine fermentation are presented in Figure 4. Throughout the fermentation, *Saccharomyces* was the most predominant genus, with its relative abundance increasing from 97.3927% in the early phase to 99.4233% in the mid phase, before slightly decreasing to 94.6363% in the late phase. In the early-fermentation phase, non-*Saccharomyces* genera were markedly enriched, such as *Lachancea* (0.9319%), *Wickerhamomyces* (0.4096%), and *Hanseniaspora* (0.5444%), along with *Clavispora* (0.0134%), *Torulaspora* (0.2897%), and the acetic acid bacterium *Gluconobacter* (0.1884%), which were enriched in trace amounts. During the mid-fermentation phase, *Limosilactobacillus* showed a notable enrichment, showing a dramatic 14,830-fold increase in abundance (from 0.00001% in the early phase to 0.1483% in the mid phase), alongside *Saccharomyces*. By the late-fermentation phase, there was an observed increase in the abundances of *Azospira* (0.0131%), *Diaphorobacter* (0.0342%), *Aquabacterium* (0.0191%), and Sphingobium (0.0385%), along with those of *Bacillus* (0.0095%), *Bifidobacterium* (0.0083%), *Parabacteroides* (0.0072%), and *Herbaspirillum* (0.0087%).

Moreover, the proportion of bacteria exhibited a progressive increase throughout the fermentation process. Specifically, the relative abundance of bacteria rose from 0.0028% in the initial phase to 0.0048% in the mid-phase, ultimately reaching 0.0525% in the late phase. Simultaneously, the yeast-to-bacteria ratio decreased from 347.82:1 in the early fermentation phase to 207.12:1 in the mid-fermentation phase and finally decreased further to 18.03:1 in the late fermentation phase.

#### 3.2.4. Analysis of Microbial Community Structure Based on Species Level

**Figure 5:**
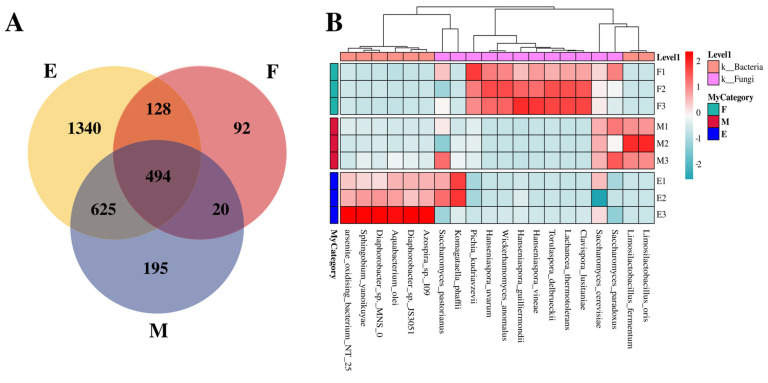
Comparative analysis of microbial community dynamics during Musalais wine fermentation. (**A**) Venn diagram illustrating phase-specific species distribution. Different colors represent different groups. The number in the overlapping area indicates the number of species shared by multiple groups, and the number in the non-overlapping area indicates the number of species unique to the corresponding group. (**B**) Heatmap profiling for relative abundance of predominant taxa. The *x*-axis represents species names, and the y-axis represents groups. The color gradient of the color blocks indicates the variation in the abundance of different species in the samples. On the right side of the figure, the color gradient representing values has been shown, and the color scale above indicates whether the species belongs to bacteria or fungi. Note: F: early-fermentation period, M: middle fermentation period, E: late-fermentation period.

In total, 2894 microbial species were identified across the three stages of Musalais wine fermentation (Figure 5A). Among them, 494 species, accounting for 17.06% of the entire microbial population, were consistently present throughout all three stages. Within this core microbiota, 24 species were non-*Saccharomyces* yeast species. These included: ① the most frequently reported yeast species, such as *Hanseniaspora vineae, Lachancea thermotolerans*, *Torulaspora delbrueckii* [25,26], *Pichia kudriavzevii* [27], *Saccharomyces bayanus*, *Hanseniaspora guilliermondii,* and *Saccharomyces paradoxus* [28,29]; ② species commonly utilized in conjunction with *Saccharomyces cerevisiae* to enhance the wine profile, including *Saccharomyces kudriavzevii*, *Saccharomyces paradoxus*, and *Schizosaccharomyces pombe* [30,31]. ③ species commonly found in other fermented foods, such as *Wickerhamomyces anomalus* [32], *Brettanomyces bruxellensis* [33,34], *Tetrapisispora phaffii*, and *Kluyveromyces marxianus* [35,36]; ④ less common species, including *Saccharomyces eubayanus* [37,38] and *Zygosaccharomyces parabailii* [39]; ⑤ and species not previously documented in fermentation processes, such as *Saccharomyces jurei*, *Sodiomyces alkalinus*, *Vanrija pseudolonga*, and *Moesziomyces antarcticus*. Notably, during the late- and early-fermentation stages, 1340 (46.30% of the total) and 92 (3.18% of the total) unique microbial species were identified.

Among the dominant species identified across various stages of fermentation (as illustrated in Figure 5B), *Saccharomyces cerevisiae* was unequivocally the most prevalent. Its relative abundance increased from 97.3484% in the early phase to 99.3769% in the mid phase, before slightly declining to 98.7919% in the late phase. Similarly, *L. thermotolerans* exhibited an increase in relative abundance from 0.0153% in the early phase to 0.9315% in the mid phase, followed by a decrease to 0.0005% in the late phase. *H. vineae* showed a rise from 0.0178% in the early phase to 0.5010% in the mid phase, before decreasing to 0.0016% in the late phase. *T. delbrueckii*’s relative abundance increased from 0.0030% in the early phase to 0.2896% in the mid phase, then decreased to 0.0002% in the late phase. *Pichia kudriavzevii* experienced an increase from 0.0295% in the early phase to 0.2709% in the mid phase, followed by a decrease to 0.0874% in the late phase. *W. anomalus* increased from 0.0013% in the early phase to 0.0407% in the mid phase, before declining to 0.0000% in the late phase. *H. guilliermondii* increased from 0.0015% during the early phase to 0.0136% in the mid phase, followed by a decline to 0.0001% in the late phase. *K. phaffii* exhibited a decrease in relative abundance from 0.0313% in the early phase to 0.004% in the mid phase, before experiencing a slight increase to 0.0047% in the late phase. Among the bacterial taxa, *Limosilactobacillus fermentum* showed an increase in relative abundance from 0.0000% in the mid phase to 0.0990% in the late phase. Similarly, *Diaphorobacter* sp. JS3051 demonstrated an initial decrease from 0.0142% in the early phase to 0.005% in the mid phase, followed by a reduction to 0.0020% in the late phase.

### 3.3. Analysis of the Correlation Between Musalais Wine Microbial Community Structure and Environmental Factors

Reportedly, the microbial community composition during fermentation is significantly affected by various environmental factors, particularly the sugar content and alcohol concentration of the fermenting substrate. Hence, the interactions between microbial community dynamics and environmental conditions of the substrate in Musalais wine fermentation were thoroughly investigated to understand the complexity of microbial ecosystems throughout the fermentation.

**Figure 6:**
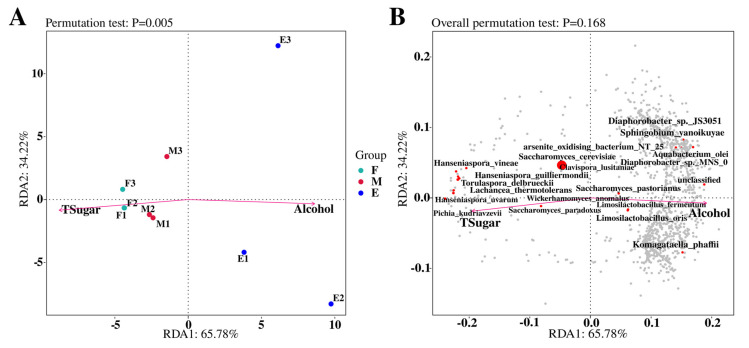
Redundancy analysis of the correlation between physicochemical parameters and core microbiota in the Musalas fermentation system. (**A**) Dynamic association of microbial community structure with environmental factors at different fermentation stages. (**B**) Species-level response of dominant genera to key physicochemical factors. Note: F: early-fermentation period, M: middle fermentation period, E: late-fermentation period.

Herein, the relative abundance of microbial communities during the early- and mid-fermentation stages of Musalais wine was positively correlated with the total sugar content and negatively correlated with the alcohol concentration of the fermenting substrate (Figure 6A). Conversely, microbial communities in the late-fermentation stage were positively and negatively correlated with alcohol and total sugar contents, respectively. Consistently, *Saccharomyce cerevisiae*, the most prevalent species within the entire microbial community, positively correlated with the total sugar content and negatively correlated with alcohol concentration (Figure 6B). *S. paradoxus* presented a similar correlation pattern. Furthermore, microorganisms that were significantly influenced by the total sugar content and showed positive correlations included *C. lusitaniae*, *T. delbrueckii*, *L. thermotolerans*, *W. anomalus*, *H. uvarum*, and *P. kudriavzevii*.

Moreover, a positive correlation was observed between bacterial abundance in Musalais wine and the ethanol content in the substrate (Figure 6B), as shown by *Diaphorobacter* sp. JS3051. Overall, these results show that during the natural fermentation of Musalais wine, microorganisms predominantly influenced by total sugar were fungi, such as *Saccharomyces spp.*, whereas those influenced by alcohol concentration were primarily bacteria. Redundancy analysis results revealed significant correlations between these physicochemical factors and microbial communities, indicating that these parameters may serve as key drivers of microbial succession during Musalais wine fermentation.

### 3.4. Correlation Analysis Among Microbial Communities in Musalais Wine

Correlations within microbial communities during Musalais wine fermentation were further explored to facilitate a deeper understanding of microbial interactions and their potential effects on both the fermentation and product quality.

**Figure 7:**
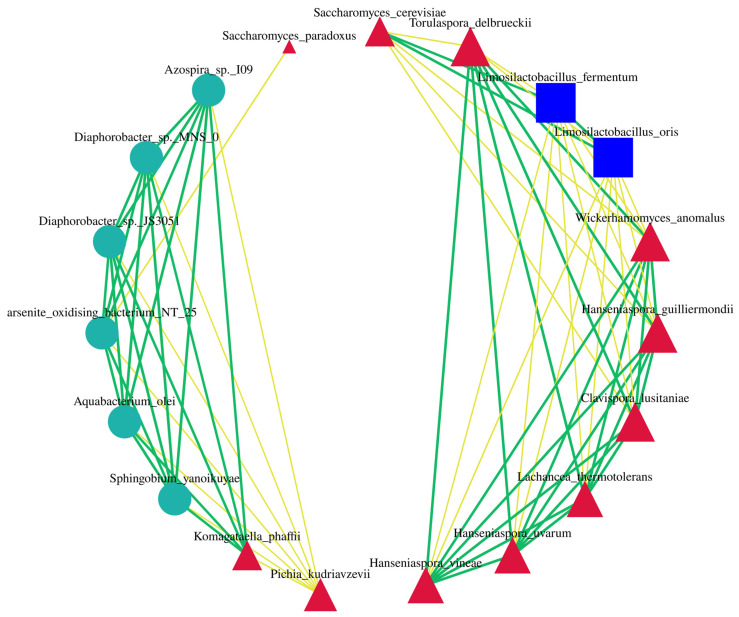
Musalais wine microbial symbiosis network diagram. Ellipses, triangles, and squares represent the phyla Pseudomonadota, Ascomycota, and Firmicutes, respectively. Yellow and green connecting lines denote negative correlations (competitive interactions) and positive correlations (mutualistic symbiosis), respectively, between microbial taxa. The area of each geometric shape corresponds to the relative abundance of the respective phylum within the microbial community.

Utilizing metagenomic sequencing data, a microbial co-occurrence network was developed with a correlation threshold of |0.05| (Figure 7) to elucidate the interaction dynamics within the microbial community during Musalais wine fermentation. Notably, *S. cerevisiae* showed significant positive correlations with *L. fermentum,* and exhibited significant negative correlations with *T. delbrueckii*, *W. anomalus*, *C. lusitaniae*, and *H. guilliermondii*. These yeast strains showed a high connectivity (degree = 9) and core topological attributes (betweenness centrality = 1). Additionally, pervasive negative correlations were identified among the yeast taxa within the phylum Ascomycota, including *T. delbrueckii*, *W. anomalus*, *C. lusitaniae*, *H. guilliermondii*, *H. uvarum*, *L. thermotolerans*, and *H. vineae*. However, these observed correlations suggest potential microbial interactions that necessitate further experimental validation.

### 3.5. Comparative Analysis of Functional Gene Enrichment in Different Fermentation Stages

**Figure 8:**
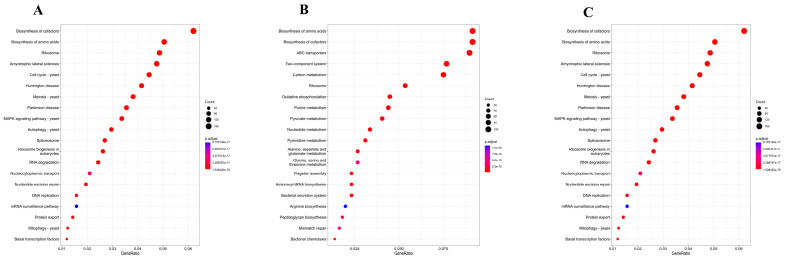
Kyoto Encyclopedia of Genes and Genomes (KEGG) pathway enrichment analysis. (**A**) Bubble chart illustrating the 20 most enriched pathways during the mid-fermentation phase relative to the early phase. (**B**) Bubble chart depicting the 20 most enriched pathways in the late-fermentation phase compared to the mid-fermentation phase. (**C**) Bubble chart representing the 20 most enriched pathways across all three fermentation phases. Statistical significance was determined at a threshold of *p* < 0.05.

The Kyoto Encyclopedia of Genes and Genomes (KEGG) enrichment analysis was conducted on genes derived from metagenomic data across various fermentation stages (Figure 8 and Supplementary Table S1). Compared to the early fermentation phase, genes from the mid-fermentation phase were predominantly enriched in the pathway of ribosome, meiosis–yeast, MAPK signaling pathway–yeast, autophagy–yeast, and DNA replication, among others. In contrast, genes from the late fermentation phase, relative to the mid phase, exhibited significant enrichment in the pathway of amino acid biosynthesis, cofactor biosynthesis, ABC transporters, oxidative phosphorylation, purine metabolism, pyruvate metabolism, and carbon metabolism, among others. Furthermore, across all three fermentation stages, functional genes demonstrated significant enrichment in 22 pathways, including carbon metabolism, ribosome, quorum sensing, propanoate metabolism, biofilm formation, pyruvate metabolism, the phosphotransferase system (PTS), sulfur relay system, flagellar assembly, and fatty acid metabolism, among others.

Gene Ontology (GO) analysis revealed that, in comparison to the early fermentation phase, the mid-fermentation phase exhibited significant upregulation of genes associated with cellular components such as the endoplasmic reticulum and Golgi apparatus, while genes related to the mitochondrial inner membrane were significantly downregulated. In the context of molecular functions, there was a pronounced upregulation of genes involved in identical protein binding, contrasted by a downregulation of genes related to DNA-binding transcription factor activity, protein kinase activity, and protein serine/threonine kinase activity. Concerning biological processes, there was a significant upregulation in the positive regulation of transcription by RNA polymerase II, accompanied by a downregulation in intracellular protein transport and vesicle-mediated transport. (Supplementary Figure S1A). As fermentation advanced, a comparative analysis of the late versus mid-fermentation phases revealed a substantial upregulation in molecular functions, such as DNA-binding transcription factor activity, protein kinase activity, and protein serine/threonine kinase activity. Additionally, there was a notable upregulation in biological processes, including intracellular protein transport and vesicle-mediated transport (Supplementary Figure S1A,B).

### 3.6. GC–MS Analysis of Volatile Compounds in Musalais Wine

**Figure 9:**
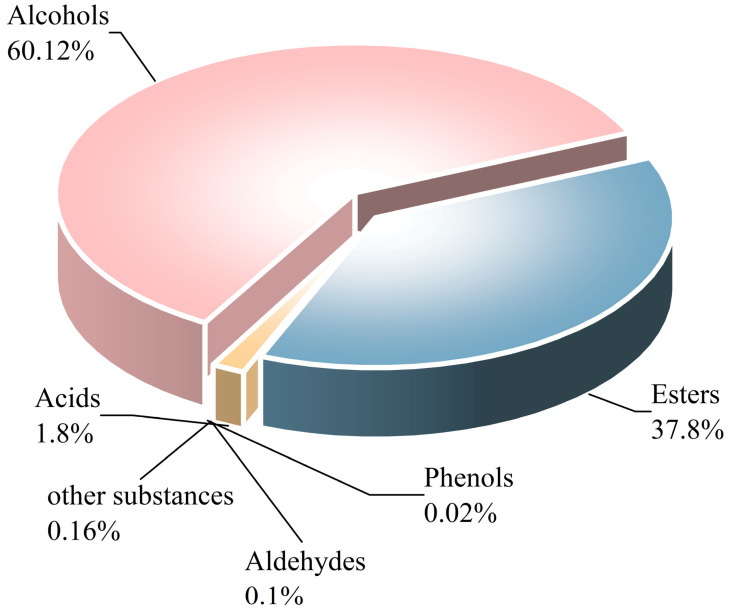
The pie chart of composition ratios of volatile components in Musalais wine per the gas chromatography–mass spectrometry analysis.

A total of 82 distinct volatile compounds were identified in spontaneously fermented Musalais wine samples (Figure 9 and Supplementary Table S2). Among them, higher alcohols, encompassing 23 compounds, had the highest total concentration. Following them, esters (39 compounds), organic acids (9 compounds), and aldehydes (5 compounds) were identified. Phenolic compounds (one compound) presented the lowest concentration.

The top four esters with the highest concentrations were ethyl octanoate (2968.05 µg/L), followed by isoamyl acetate (2381.30 µg/L), phenethyl acetate (1831.55 µg/L), and ethyl decanoate (1079.85 µg/L) (Supplementary Table S2). The top three higher alcohols with the highest concentrations were isoamyl alcohol (6513.70 µg/L), 3-methylbutanol (6042.90 µg/L), and phenethyl alcohol (2083.35 µg/L). The top three aldehydes with the highest concentrations were acetaldehyde (8.75 µg/L), nonanal (7.15 µg/L), and decanal (7.05 µg/L). Notably, nonanal is recognized for contributing sweet orange and rose floral notes, and decanal imparts a subtle spicy aroma. The fatty acids with the highest concentrations were octanoic acid (213.4 µg/L) and decanoic acid (121.15 µg/L). Additionally, trace amounts of terpenoids were detected, including citronellol (14.25 µg/L), linalool (5.45 µg/L), trans-nerolidol (4.50 µg/L), nerol (0.90 µg/L), and α-terpineol (0.60 µg/L).

## 4. Discussion

During the natural fermentation of Musalais wine, the composition and dynamic changes of the microbial community considerably affect the flavor and quality of the final product. A thorough analysis of the dynamic changes and interactions of the microbial community during the natural fermentation of Musalais wine can provide a theoretical basis for optimizing the fermentation of Musalais wine and improving product quality.

### 4.1. Microbial Origins in Musalais Wine: Resilience Beyond Heat Sterilization

The production process of Musalais wine includes a thermal concentration phase, a practice presumably derived from historical limitations associated with grape maturity, which has been sustained over time [1,5]. During this thermal concentration, the sugar content of the grape must increase, which consequently augments the alcohol content in the final product. During this phase, the indigenous microbial population within the grape is eradicated, followed by the effective initiation of spontaneous fermentation in the cooled, concentrated must. This suggests that microbial recolonization occurs from external sources, including the equipment and the surrounding environment.

These microbial communities present in the environment are not randomly distributed; rather, they originate from the grape itself [40,41]. When grapes are crushed and handled, microbial cells, including yeasts and bacteria, are introduced into the environment, subsequently colonizing equipment and surfaces. Hence, a reservoir of grape-derived microbes is established through which the functional taxa are reintroduced into the thermally concentrated must. Herein, *S*. *cerevisiae* comprised 94.64–99.42% of the total microbial population throughout the entire fermentation. This observation contrasts with findings from other studies, which suggest that *S. cerevisiae* typically predominates during the late-fermentation stage, whereas other yeasts and bacteria are more prevalent in the early stages [10]. This discrepancy may be attributed to the higher survival rate of *S. cerevisiae* in winery environments [11]. Furthermore, consistent with previous studies [42], non-*Saccharomyces* yeasts, including *Hanseniaspora* and *Torulaspora*, were found to be present during various stages of Musalais wine fermentation. These yeasts are typically found on grape skins; they persist in processing environments and subsequently play a significant role in driving fermentation.

### 4.2. Dynamic Changes in Functional Gene Enrichment During the Fermentation Process and Identification of Key Pathways

In the context of spontaneous fermentation, the evolution of microbial communities and their metabolic pathways are crucial for the formation of flavor compounds. Analyses of metagenomic data using KEGG and GO frameworks have elucidated the metabolic dynamics of fermentation-associated microbial communities at the molecular level [43,44]. KEGG analysis indicates a notable enrichment of pathways such as ribosome and MAPK signaling during mid-fermentation, indicative of active cellular processes [45]. In the late fermentation stage, pathways associated with amino acid and cofactor biosynthesis, as well as pyruvate and carbon metabolism, were enriched, reflecting a metabolic shift in yeast [46,47]. Across all stages, consistent gene enrichment was observed in pathways including carbon metabolism, ribosome, and fatty acid metabolism, underscoring their essential roles in the fermentation process.

GO analysis reveals that during the mid-fermentation phase, there was an upregulation of endoplasmic reticulum-related genes, likely due to increased protein synthesis and processing needs [48], while genes associated with the mitochondrial inner membrane genes, suggesting reduced energy demands or altered energy pathways [49]. During late fermentation, cells upregulated DNA-binding transcription factors to adapt to nutrient scarcity and metabolic product accumulation by activating specific genes [50]. Increased protein kinase and serine/threonine kinase activities suggest the activation of signaling pathways in response to environmental stress, cell cycle regulation, and structural remodeling [51]. The increased intracellular and vesicle-mediated protein transport suggests a higher demand for protein sorting within the cell, likely due to needs for protein synthesis, folding, modification, and environmental responses [52].

### 4.3. Microbial Diversity as a Catalyst for Volatile Compounds

Herein, 82 distinct volatile compounds were identified in Musalais wine samples, with higher alcohols, esters, and organic acids being the most prominent. These results are consistent with the findings of LX Zhu et al. [1]. The inherent extensive microbial diversity in Musalais wine is closely related to its distinctive volatile profile. Studies have shown that *Saccharomyces, Hanseniaspora, Zygosaccharomyces*, and others are the dominant microbial groups in spontaneous fermentation. These microbial groups are significantly positively correlated with flavor compounds such as organic acids, fatty acids, esters, phenols, aldehydes, and terpenes [53]. Reportedly, the synergistic interactions among different microorganisms during spontaneous fermentation significantly affect the metabolism and the production of aroma precursors, thereby enhancing the wine’s aromatic complexity. During spontaneous fermentation, mixed fermentations involving *S. kudriavzevii* and *S. cerevisiae* can result in increased glycerol levels. In such mixed fermentations, indigenous *S. cerevisiae* strains have been shown to facilitate the survival of *P. kudriavzevii,* thereby augmenting the content of fruity esters and terpenes [54]. Additionally, mixed fermentations with *S. paradoxus* with *S. cerevisiae* have been shown to lead to higher concentrations of alcohols and reduced levels of L-malate and sulfites [55].

Non-*Saccharomyces* yeasts play a pivotal role in shaping the flavor and sensory attributes of wine. For instance, *H. guilliermondii* can influence wine aroma by forming esters and higher alcohols [56]. *T. delbrueckii* exhibits significant beta-lyase activity [27], which has been shown to positively impact the aromatic profile. *H. uvarum* can lead to an increased alcohol content and enhanced fruity notes [57]. *L. thermotolerans* can increase the production of 2-phenylethyl alcohol and 2-phenylethyl acetate, which have been associated with “rose”, “fruity”, and “floral” aromas in wine [58]. *P. kudriavzevii* enhances the profile of flavor compounds such as ethyl acetate [27]. *T. delbrueckii* can enhance fruity aromas and accelerate malolactic fermentation [25,26]. *S. bayanus* has been shown to produce volatile compounds, including the high-concentration ethyl esters, isoamyl acetate, 2-phenylethyl acetate, ethyl palmitate, and hexanol [59]. *B. bruxellensis* reduces alcohol content while increasing the levels of individual phenolics.

Other than yeast, some bacteria, such as *Oenococcus oeni* and *Lactiplantibacillus plantarum*, have been reported to play a crucial role in dominating malolactic fermentation in wine. These strains facilitate the production of glycosidases that further enhance the wine’s aroma [60]. Furthermore, *L. fermentum* exhibits a high glycoside hydrolase activity, which has been shown to contribute to the aromatic profile of wine [61]. *Lacticaseibacillus casei* has demonstrated superior activity in producing ethyl hexanoate and ethyl octanoate, and *Lactobacillus helveticus* can produce a high content of ethyl acetate [62]. Additionally, non-*Saccharomyces* yeasts and bacteria can interact synergistically to produce volatile compounds.

### 4.4. Microbial Diversity and Associated Health Issues in Spontaneous Fermentation

Musalais wine exhibits significant microbial diversity, the exploration and preservation of which are essential for sustaining traditional Musalais wine practices. A thorough analysis of each microbe’s role in wine fermentation and its impact on sensory quality can provide a comprehensive understanding of the aromatic contributions of individual microbes. Furthermore, investigating the fermentation properties of previously unexamined microorganisms within the contexts, such as *Saccharomyces jurei*, *Sodiomyces alkalinus*, *Vanrija pseudolonga*, and *Moesziomyces antarcticus*, of wine and food fermentation could provide valuable insights for the development of novel microbial strains.

This process, however, may introduce harmful microorganisms that could compromise both the quality and safety of the product [63]. Similar to other naturally fermented products, we have identified the presence of potential pathogens, including Brettanomyces bruxellensis, a common contaminant in alcoholic beverages known for imparting undesirable flavors and increasing volatile acidity, thus compromising product quality [64]. Fortunately, the relative abundance of these microorganisms is minimal. Managing harmful microorganisms during the natural fermentation of alcoholic beverages presents a complex yet crucial challenge [65,66]. Conducting comprehensive research on the composition and dynamics of microbial communities, screening and isolating beneficial fermentative microorganisms, and developing mixed fermentation agents are of significant importance for maintaining the flavor of Musalais wine and ensuring its food safety.

### 4.5. Microbial Diversity from Natural Fermentation in Musalais Wine and Its Importance for Building a Microbial Resource Bank

Natural fermentation represents a complex biological process characterized by the interaction of diverse microorganisms, which are essential in determining the flavor and quality of Musalais. To enhance the understanding and application of microbial diversity during fermentation, the establishment and maintenance of microbial resource banks are of paramount importance. Moreover, preserving microbial diversity in natural fermentation processes is critical for sustaining the flavor and quality of traditional foods [67]. This diversity not only influences the flavor profile of food but may also impact its nutritional value and safety.

Importantly, since the late 19th century, microbial culture collections have been indispensable to the field of microbiology, significantly contributing to scientific advancement through the preservation and distribution of microbial strains [68]. These repositories are crucial not only for supporting biotechnology, personalized medicine, agriculture, and environmental sustainability but also as essential instruments for public education, helping to dispel misconceptions about microbes. Therefore, establishing microbial resource banks for Musalais wine is crucial for enhancing natural fermentation processes and for the conservation and utilization of microbial diversity.

## 5. Conclusions

Herein, a comprehensive analysis was conducted to investigate the microbial community dynamics and volatile compounds involved in the natural fermentation of Musalais wine. The findings revealed that the microbial community structure undergoes substantial changes across various fermentation stages, with distinct microbial compositions characterizing each phase. In total, 2894 microbial species were identified across the three fermentation stages, with 494 species detected across all stages. The late-fermentation stage presented with the highest diversity of unique microbial species (46.30% of the total microbial population). *S. cerevisiae* was found to be the predominant species throughout the fermentation; however, various non-*Saccharomyces* yeasts and bacteria also seemed to make vital contributions. Notably, 79 microbial taxa exhibiting significant variations during fermentation were identified. Specifically, differential genera such as *Valsa*, *Hanseniaspora*, and *Pichia* were found to be prominent in the early stage; *Limosilactobacillus* was notable in the mid stage; and many genera, including *Brevibacterium*, *Kytococcus*, and *Ornithinimicrobium*, were determined to be prominent in the late stage.

The investigation into the volatile compounds of Musalais wine identified 82 distinct compounds, with higher alcohols, esters, and organic acids being the most predominant. This research provides a theoretical basis for the preservation and sustainable development of Musalais wine. However, the direct relationship between microbial activity and the volatile profile of Musalais wine necessitates further empirical investigation for validation.

## Data Availability

The original contributions presented in this study are included in the article/Supplementary Material. Further inquiries can be directed to the corresponding author.

## Supplementary Materials

The following supporting information can be downloaded at: https://www.mdpi.com/article/10.3390/foods14152570/s1, Supplementary Table S1. KEGG classifications of the metagenome across the three fermentation phases of Musalais wine. Note: F vs. M represents the statistically significant differences in KEGG pathways between the mid-fermentation phase and the early fermentation stage. M vs. E indicates the significant differences in KEGG pathways between the late-fermentation phase and the mid-fermentation stage. The intersection KEGG pathways across all three stages represents the significantly enriched KEGG pathways across all three fermentation stages. Statistical significance was determined at a threshold of *p* < 0.05. Supplementary Figure S1. GO enrichment analysis of identified genes in metagenomics. Based on functions, these are classified into three groups (Component: cellular components; Functions: molecular functions, and Processes: biological processes). (A) Significant differences in gene expression ralated to GO terms between the mid-fermentation phase and the early fermentation stage. (B) Significant differences in GO terms were observed between the late-fermentation phase and the mid-fermentation stage. Supplementary Table S2. Volatile composition and content of Musalais wines. Supplementary Table S3. Statistics of foundational data prior to and following the filtration process.
