# Supplementary material for: Microbial Dynamics in a Musalais Wine Fermentation: A Metagenomic Study
Source: Foods. 2025 Jul 22;14(15):2570. doi: 10.3390/foods14152570 (PMC12346735; doi:10.3390/foods14152570)
Supplement: Supplementary file 1 [file foods-14-02570-s001.zip › foods-3714795-supplementary 1.pdf]

Supplementary Table S1. KEGG classifications of the metagenome across the three fermentation phases of Musalais. Note: F vs. M represents the statistically significant differences in KEGG pathways between the mid-fermentation phase and the early fermentation stage. M vs. E indicates the significant differences in KEGG pathways between the late-fermentation phase and the mid-fermentation stage. The intersection KEGG pathways across all three stages represents the significantly enriched KEGG pathways across all three fermentation stages. Statistical significance was determined at a threshold of $p<0.05$.

	ID	Description	pvalue	qvalue
	ko00100	Steroid biosynthesis	0.00	0.00
	ko01212	Fatty acid metabolism	0.00	0.00
	ko00400	Phenylalanine, tyrosine and tryptophan biosynthesis	0.00	0.00
	ko05110	Vibrio cholerae infection	0.00	0.00
	ko04966	Collecting duct acid secretion	0.00	0.00
	ko00790	Folate biosynthesis	0.00	0.00
	ko00780	Biotin metabolism	0.00	0.00
	ko04150	mTOR signaling pathway	0.00	0.00
	ko04721	Synaptic vesicle cycle	0.00	0.00
	ko04112	Cell cycle - Caulobacter	0.00	0.00
	ko00520	Amino sugar and nucleotide sugar metabolism	0.00	0.00
	ko03450	Non-homologous end-joining	0.00	0.00
	ko05208	Chemical carcinogenesis - reactive oxygen species	0.00	0.00
	ko00480	Glutathione metabolism	0.00	0.00
	ko00983	Drug metabolism - other enzymes	0.00	0.00
	ko00740	Riboflavin metabolism	0.00	0.00
	ko04137	Mitophagy - animal	0.00	0.00
F vs. M	ko00061	Fatty acid biosynthesis	0.00	0.01
	ko04070	Phosphatidylinositol signaling system	0.00	0.01
	ko04145	Phagosome	0.00	0.01
	ko00521	Streptomycin biosynthesis	0.00	0.01
	ko00670	One carbon pool by folate	0.00	0.01
	ko00500	Starch and sucrose metabolism	0.00	0.01
	ko04213	Longevity regulating pathway - multiple species	0.00	0.01
	ko01040	Biosynthesis of unsaturated fatty acids	0.00	0.01
	ko00030	Pentose phosphate pathway	0.01	0.02
	ko00562	Inositol phosphate metabolism	0.01	0.02
	ko00550	Peptidoglycan biosynthesis	0.01	0.02
	ko00410	beta-Alanine metabolism	0.01	0.02
	ko01524	Platinum drug resistance	0.01	0.02
	ko00785	Lipoic acid metabolism	0.01	0.02
	ko03460	Fanconi anemia pathway	0.01	0.02
	ko00760	Nicotinate and nicotinamide metabolism	0.01	0.03
	ko00561	Glycerolipid metabolism	0.01	0.04
	ko04914	Progesterone-mediated oocyte maturation	0.01	0.04

	ko04922	Glucagon signaling pathway	0.01	0.04
	ko00630	Glyoxylate and dicarboxylate metabolism	0.02	0.05
	ko00600	Sphingolipid metabolism	0.02	0.05
	ko04260	Cardiac muscle contraction	0.02	0.06
	ko04666	Fc gamma R-mediated phagocytosis	0.02	0.07
	ko04981	Folate transport and metabolism	0.02	0.07
	ko00450	Selenocompound metabolism	0.03	0.08
	ko03070	Bacterial secretion system	0.03	0.08
	ko00330	Arginine and proline metabolism	0.03	0.09
	ko02030	Bacterial chemotaxis	0.04	0.12
M vs. E	ko01503	Cationic antimicrobial peptide (CAMP) resistance	0.00	0.00
	ko00330	Arginine and proline metabolism	0.00	0.00
	ko00910	Nitrogen metabolism	0.00	0.00
	ko00730	Thiamine metabolism	0.00	0.00
	ko00785	Lipoic acid metabolism	0.00	0.00
	ko00983	Drug metabolism - other enzymes	0.00	0.00
	ko00660	C5-Branched dibasic acid metabolism	0.00	0.01
	ko00480	Glutathione metabolism	0.00	0.01
	ko02025	Biofilm formation - Pseudomonas aeruginosa	0.00	0.01
	ko00750	Vitamin B6 metabolism	0.00	0.01
	ko00052	Galactose metabolism	0.00	0.01
	ko03018	RNA degradation	0.00	0.01
	ko02060	Phosphotransferase system (PTS)	0.00	0.02
	ko00130	Ubiquinone and other terpenoid-quinone biosynthesis	0.00	0.02
	ko00900	Terpenoid backbone biosynthesis	0.00	0.02
	ko01250	Biosynthesis of nucleotide sugars	0.01	0.02
	ko00790	Folate biosynthesis	0.01	0.02
	ko02026	Biofilm formation - Escherichia coli	0.01	0.03
	ko00410	beta-Alanine metabolism	0.01	0.03
	ko01310	Nitrogen cycle	0.01	0.04
	ko00907	Pinene, camphor and geraniol degradation	0.01	0.04
	ko00051	Fructose and mannose metabolism	0.02	0.07
	ko00521	Streptomycin biosynthesis	0.02	0.07
	ko00740	Riboflavin metabolism	0.03	0.08
	ko00930	Caprolactam degradation	0.03	0.08
	ko01502	Vancomycin resistance	0.03	0.08
	ko00791	Atrazine degradation	0.04	0.10
	ko00350	Tyrosine metabolism	0.04	0.11
	ko04981	Folate transport and metabolism	0.04	0.11
	ko05230	Central carbon metabolism in cancer	0.04	0.11
Intersection KEGG pathways across all	ko01200	Carbon metabolism	0.00	0.00
	ko05111	Biofilm formation - Vibrio cholerae	0.00	0.00
	ko04122	Sulfur relay system	0.00	0.00
	ko00720	Other carbon fixation pathways	0.00	0.00

three stages	ko02060	Phosphotransferase system (PTS)	0.00	0.00
	ko03070	Bacterial secretion system	0.00	0.00
	ko00620	Pyruvate metabolism	0.00	0.00
	ko02024	Quorum sensing	0.00	0.00
	ko02040	Flagellar assembly	0.00	0.02
	ko00907	Pinene, camphor and geraniol degradation	0.01	0.04
	ko00430	Taurine and hypotaurine metabolism	0.01	0.04
	ko00660	C5-Branched dibasic acid metabolism	0.01	0.04
	ko01212	Fatty acid metabolism	0.01	0.04
	ko04112	Cell cycle - Caulobacter	0.01	0.04
	ko00650	Butanoate metabolism	0.02	0.08
	ko00440	Phosphonate and phosphinate metabolism	0.03	0.08
	ko03060	Protein export	0.03	0.08
	ko01232	Nucleotide metabolism	0.03	0.08
	ko00270	Cysteine and methionine metabolism	0.03	0.08
	ko00071	Fatty acid degradation	0.04	0.10
	ko01230	Biosynthesis of amino acids	0.04	0.10
	ko00053	Ascorbate and aldarate metabolism	0.05	0.10

Supplementary Figure S1. GO enrichment analysis of identified genes in metagenomics. Based on functions, these are classified into three groups (Component: cellular components; Functions: molecular functions, and Processes: biological processes). (A) Significant differences in gene expression ralated to GO terms between the mid-fermentation phase and the early fermentation stage. (B) Significant differences in GO terms were observed between the late-fermentation phase and the mid-fermentation stage.

A

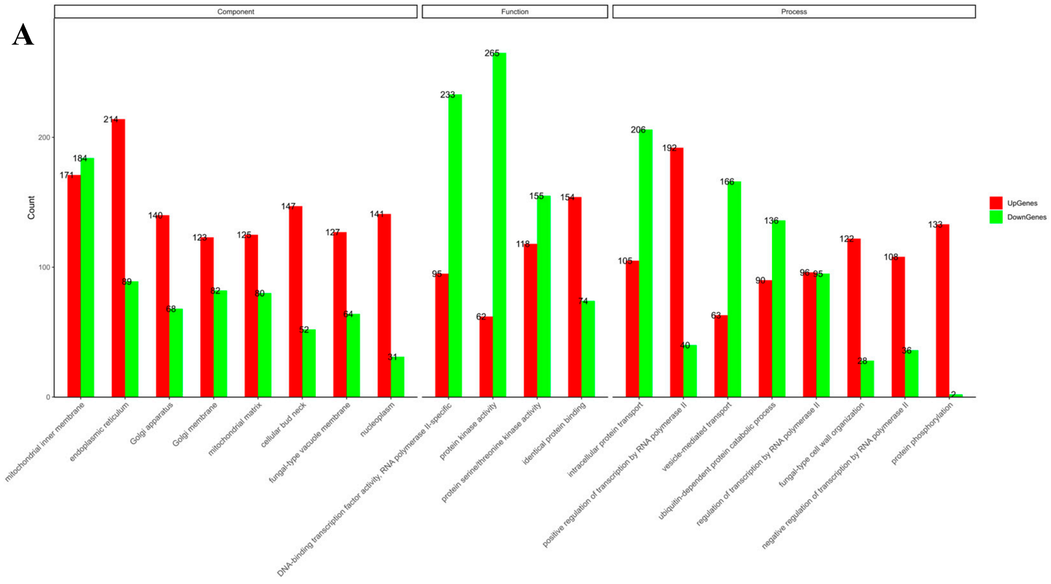

B

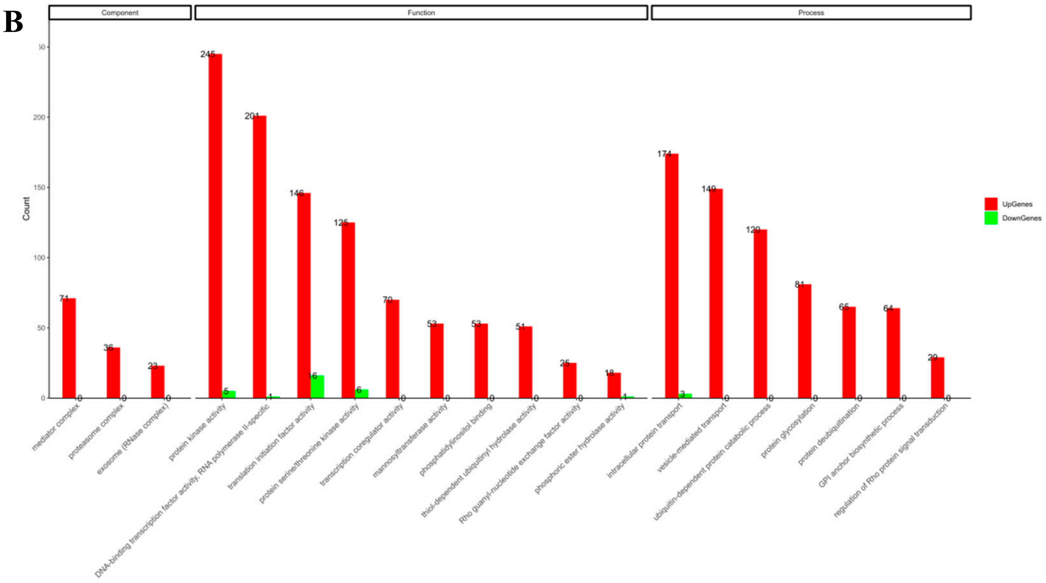

Component
Function
Process
Count
200
150
100
50
0
UpGenes
DownGenes
71
36
23
245
201
146
125
70
53
53
51
25
18
174
149
120
81
65
64
29
5
1
16
6
1
3
mediator complex
proteasome complex
exosome (RNase complex)
protein kinase activity
DNA-binding transcription factor activity, RNA polymerase II-specific
translation initiation factor activity
protein serine/threonine kinase activity
transcription coregulator activity
mannosyltransferase activity
phosphatidylinositol binding
thiol-dependent ubiquitinyl hydrolase activity
Rho guanyl-nucleotide exchange factor activity
phosphoric ester hydrolase activity
intracellular protein transport
vesicle-mediated transport
ubiquitin-dependent protein catabolic process
protein glycosylation
protein deubiquitination
GPI anchor biosynthetic process
regulation of Rho protein signal transduction

Supplementary Table S2. Aroma composition and content of Musalais wines

NO.	Aroma category	Aroma compounds	Content(μg/L)
1	Ethyl esters	Ethyl acetate	229.90
2		Ethyl hexanoate	376.50
3		Ethyl butyrate	15.15
4		Ethyl lactate	8.85
5		Ethyl decanoate	1079.85
6		Ethyl octanoate	2968.05
7		Ethyl 7-octenoate	5.10
8		Ethyl 9-decenoate	471.20
9		Ethyl phenylacetate	3.80
10		Ethyl laurate	183.10
11		Ethyl nonanoate	4.75
12		Ethyl 3-(methylthio)propionate	2.85
13	Acetate esters	Hexyl acetate	3.00
14		Isoamyl acetate	2381.30
15		Isobutyl acetate	13.70
16		Phenethyl acetate	1831.55
17		Octyl acetate	9.25
18		Citronellyl acetate	7.65
19		Geranyl acetate	2.60
20	Other esters	Methyl salicylate	2.60
21		Isopentyl caprylate	9.25
22		Ethyl 3-methylbutyl succinate	8.60
23		Ethyl 9-hexadecenoate	2.45
24		Ethyl palmitate	7.00
25		Isopentyl propionate	11.40
26		Isopentyl butyrate	1.80
27		Ethyl phenylpropionate	3.80
28		Ethyl 8-methylnonanoate	6.50

NO.	Aroma category	Aroma compounds	Content(μg/L)
29		Phenethyl octanoate	1.20
30		Isopentyl hexanoate	8.15
31		Methyl decanoate	1.10
32		Isopentyl caprylate	23.60
33		Methyl nonyl carbonate	26.50
34		Methyl palmitate	1.80
35		β -Phenethyl butyrate	3.20
36		*n*-hexyl salicylate	1.30
37		Isopentyl lactate	2.75
38		3-Methylbutyl octanoate	25.80
39		Diethyl succinate	39.75
40		2-Methylpropanol	88.05
41		*n*-Butanol	8.90
42		2-Methylbutanol	652.30
43		Isoamyl alcohol	6513.70
44		4-Penten-1-ol	3.80
45		4-Methyl-1-pentanol	9.85
46		3-Methyl-1-pentanol	20.80
47		2-Ethylhexanol	7.45
48	**Higher Alcohols**	1-Octanol	31.60
49		1-Decanol	31.65
50		Lauryl alcohol	17.35
51		Myristyl alcohol	8.15
52		DL-2,3-Dihydro-6-trans-farnesol	4.40
53		Phenethyl alcohol	2083.35
54		2-Nonanol	1.60
55		3-Methylbutanol	6042.90
56		3-(Methylthio)-1-propanol	8.85
57	**Terpenes**	Linalool	5.45

NO.	Aroma category	Aroma compounds	Content(μg/L)
58		α-Terpineol	0.60
59		Citronellol	14.25
60		trans-Nerolidol	4.50
61		Farnesol	3.10
62		Nerolidol	0.90
63	**Phenols**	4-Vinylguaiacol	5.40
64		Acetaldehyde	8.75
65		Nonanal	7.05
66	**Aldehydes**	Decanal	7.15
67		Benzaldehyde	2.40
68		5-Methylfurfural	1.95
69		1,2-Dimethylcyclopent-2-enecarboxylic	17.65
70		Acetic acid	19.00
71		Hexanoic acid	29.40
72		Octanoic acid	213.40
73	**Organic Acids**	*n*-Decanoic acid	121.15
74		9-Decenoic acid	15.55
75		Lauric acid	14.10
76		3-(2,2,4-Trimethylcyclohex-3-enyl)propenoic acid	32.15
77		4-Hydroxybutyric acid	2.45
78		2,3-Dimethylpentane	24.70
79		Furfuryl ethyl ether	2.80
80	**Others**	1,2-Dimethylcyclopentane	9.35
81		2-Acetylfuran	2.60
82		Phyketone	1.35
