# Supplementary material for: Microbial Dynamics in a Musalais Wine Fermentation: A Metagenomic Study
Source: Foods. 2025 Jul 22;14(15):2570. doi: 10.3390/foods14152570 (PMC12346735; doi:10.3390/foods14152570)
Supplement: Supplementary file 1 [file foods-14-02570-s001.zip › foods-3714795-supplementary 2.pdf]

Supplementary Table S3. Statistics of foundational data prior to and following the filtration process.

Sample ID	InsertSize(bp)	SeqStrategy	RawReads	Raw Base(GB)	%GC	Raw Q20(%)	Raw Q30(%)	Clean Reads	Cleaned(%)	Clean Q20(%)	Clean Q30(%)
**E1**	350	(150:150)	37012200	11.1	39	99.21	97.47	35770929	96.65	99.58	98.34
**E2**	350	(150:150)	20506738	6.15	41	98.55	96.08	18704119	91.21	99.64	98.57
**E3**	350	(150:150)	21602310	6.48	39	99.04	96.96	20678612	95.72	99.49	98.01
**F1**	350	(150:150)	21735278	6.52	38	99.25	97.59	21081520	96.99	99.6	98.42
**F2**	350	(150:150)	24568647	7.37	38	99.23	97.55	23788905	96.83	99.59	98.38
**F3**	350	(150:150)	26546639	7.96	38	99.23	97.54	25760632	97.04	99.58	98.37
**M1**	350	(150:150)	28146357	8.44	38	99.24	97.56	27267381	96.88	99.59	98.39
**M2**	350	(150:150)	29296768	8.79	38	99.24	97.55	28352598	96.78	99.58	98.35
**M3**	350	(150:150)	29455437	8.84	38	99.22	97.5	28516574	96.81	99.58	98.36
